# Social Costs of Iron Deficiency Anemia in 6–59-Month-Old Children in India

**DOI:** 10.1371/journal.pone.0136581

**Published:** 2015-08-27

**Authors:** Rafael Plessow, Narendra Kumar Arora, Beatrice Brunner, Christina Tzogiou, Klaus Eichler, Urs Brügger, Simon Wieser

**Affiliations:** 1 Winterthur Institute of Health Economics, Zurich University of Applied Sciences, Winterthur, Switzerland; 2 International Clinical Epidemiology Network, INCLEN, New Delhi, India; University College London, UNITED KINGDOM

## Abstract

**Introduction:**

Inadequate nutrition has a severe impact on health in India. According to the WHO, iron deficiency is the single most important nutritional risk factor in India, accounting for more than 3% of all disability-adjusted life years (DALYs) lost. We estimate the social costs of iron deficiency anemia (IDA) in 6–59-month-old children in India in terms of intangible costs and production losses.

**Materials and Methods:**

We build a health economic model estimating the life-time costs of a birth cohort suffering from IDA between the ages of 6 and 59 months. The model is stratified by 2 age groups (6–23 and 24–59-months), 2 geographical areas (urban and rural), 10 socio-economic strata and 3 degrees of severity of IDA (mild, moderate and severe). Prevalence of anemia is calculated with the last available National Family Health Survey. Information on the health consequences of IDA is extracted from the literature.

**Results:**

IDA prevalence is 49.5% in 6–23-month-old and 39.9% in 24–58-month-old children. Children living in poor households in rural areas are particularly affected but prevalence is high even in wealthy urban households. The estimated yearly costs of IDA in 6–59-month-old children amount to intangible costs of 8.3 m DALYs and production losses of 24,001 m USD, equal to 1.3% of gross domestic product. Previous calculations have considerably underestimated the intangible costs of IDA as the improved WHO methodology leads to a threefold increase of DALYs due to IDA.

**Conclusion:**

Despite years of iron supplementation programs and substantial economic growth, IDA remains a crucial public health issue in India and an obstacle to the economic advancement of the poor. Young children are especially vulnerable due to the irreversible effects of IDA on cognitive development. Our research may contribute to the design of new effective interventions aiming to reduce IDA in early childhood.

## Introduction

Inadequate nutrition has a severe impact on health in India. According to the WHO Global Burden of Disease project (GBD), 6 out of the leading 15 health risk factors in India are related to inadequate nutrition and are responsible for more than 18% of all Disability Adjusted Life Years (DALYs) lost [1].

The single most important nutritional risk factor in India is iron deficiency, with more than 3% of all DALYs lost [1]. Iron deficiency anemia (IDA) is highly prevalent among Indian children in spite of substantial economic growth and numerous programs aimed at the reduction of anemia [2]. Iron deficiency in early childhood is especially detrimental due to increased mortality and its permanent impact on cognitive development, which leads to an irreversible loss of productivity in adult life [3].

This paper assesses the social costs of IDA in 6–59-month-old children in India in 2013. These social costs have 2 dimensions: 1) The human burden in terms of life years lost due to premature death and in terms of quality of life lost due to morbidity and impaired cognitive development (both measured in DALYs). 2) The economic costs in terms of lower productivity due to impaired cognitive development leading to lower income in adulthood (measured in monetary losses).

We consider reversible as well as irreversible health consequences of IDA in 2 age sub-groups (6–23 and 24–59-month-old) and assess the distribution of the consequent social costs across 10 socio-economic strata (SES) and 2 geographical areas (urban and rural). The distinction between age groups is important because the health consequences and the prevalence of IDA may change with age. The 6‐23‐month-period is part of the “first 1000 days” period from conception to the second birthday, which has been identified as a crucial period to establish a lasting foundation for health [4]. Nutrition is also likely to differ between the 2 age groups. The distinction between urban and rural households and socio-economic groups is important because the economic and social conditions are likely to affect the levels of IDA in children. Although the majority of Indians still live in rural areas, the urban population is now at around 400 million (m) and rapidly growing [5]. The urban poor may be particularly affected by IDA as they live in unhealthy environmental conditions and are not reached by the same social programs as the rural poor [6, 7].

Furthermore, we examine an important methodological issue: The GBD project has recently developed a new methodology and new disability weights for the calculation of DALY losses [8], which may lead to substantial changes in the measurement of the burden of IDA. We compare the burden of IDA according to the old and to the new methodology, a comparison that has not been done before.

## Methods

**Fig 1:**
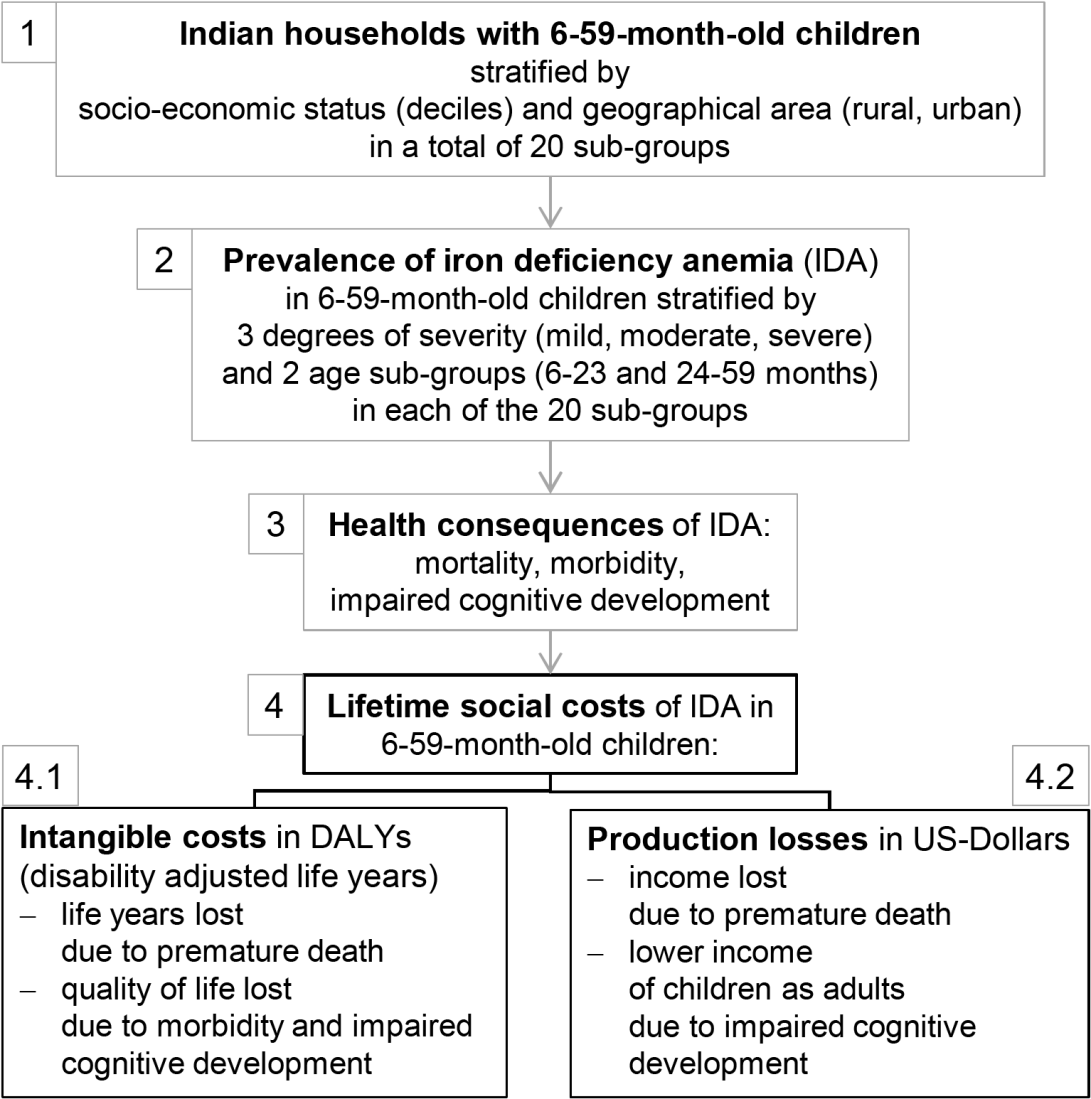
Overview of health economic model of social costs of IDA. Adapted from Wieser, Plessow [9].

Our study is based on a health economic model estimating the lifetime health and cost consequences of IDA in 6–59-month-old children which we developed in Wieser et al. [9]. Fig 1 gives an overview of the model structure.

In a first step we stratify Indian households with 6–59-month-old children by SES and geographical area (urban, rural). For this we use the National Family Health Survey of 2005–06 (NFHS-3) [10] and 2011 Census data [5], which are the most recent data available. The NFHS-3 is a large, representative population survey that provides information on 109,041 households in India. The 10 SES are built upon the distribution of households across the 10 deciles of a wealth index calculated according to the Demographic and Health Survey (DHS) methodology [11].

In a second step we calculate the prevalence of mild, moderate and severe IDA in 2 age groups (6–23 and 24–59-month-old). Prevalence of IDA is calculated according to the altitude-adjusted hemoglobin (Hb) values of the NFHS-3 [10] and assuming a normal distribution defined by the mean and the standard deviations (SD) of blood Hb. Although the NFHS-3 data were collected in 2005–06, there is no indication that the prevalence of anemia in India has decreased in the meantime. Prevalence of anemia actually increased between 1998–99 (NFHS-2) and 2005–06 (NFHS-3) and recent studies find comparable prevalence rates [12, 13].

Anemia may have other causes besides iron deficiency and the share attributable to iron deficiency varies according to age group and region. Based on a recent systematic review [14] and a WHO report [15], we attribute 60% of cases of anemia in 6–59-month-old Indian children to iron deficiency.

The 3 degrees of severity of anemia are defined according to WHO thresholds for children under 5 (mild anemia: Hb < 110 g/l, moderate anemia: Hb < 100 g/l, severe anemia: Hb <70 g/l) [16]. We smooth the distribution of the prevalence of IDA across SES with a linear model in order to remove the sample noise when extrapolating the prevalence of IDA to the whole population.

In a third step we attribute specific health consequences to IDA at different levels of severity in the 2 age sub-groups. This attribution of health consequences is mainly based on information derived from systematic reviews of iron supplementation trials. We assume that iron supplementation fills the gap of iron intake in iron-deficient children and thus eliminates all the adverse health consequences of IDA, an approach also applied in previous research [9, 17]. The health consequences of IDA thus correspond to the difference in health status of the intervention and the control group in the supplementation trials. The influence of IDA on mortality is modeled as an attributable fraction of all-cause mortality.

**Fig 2:**
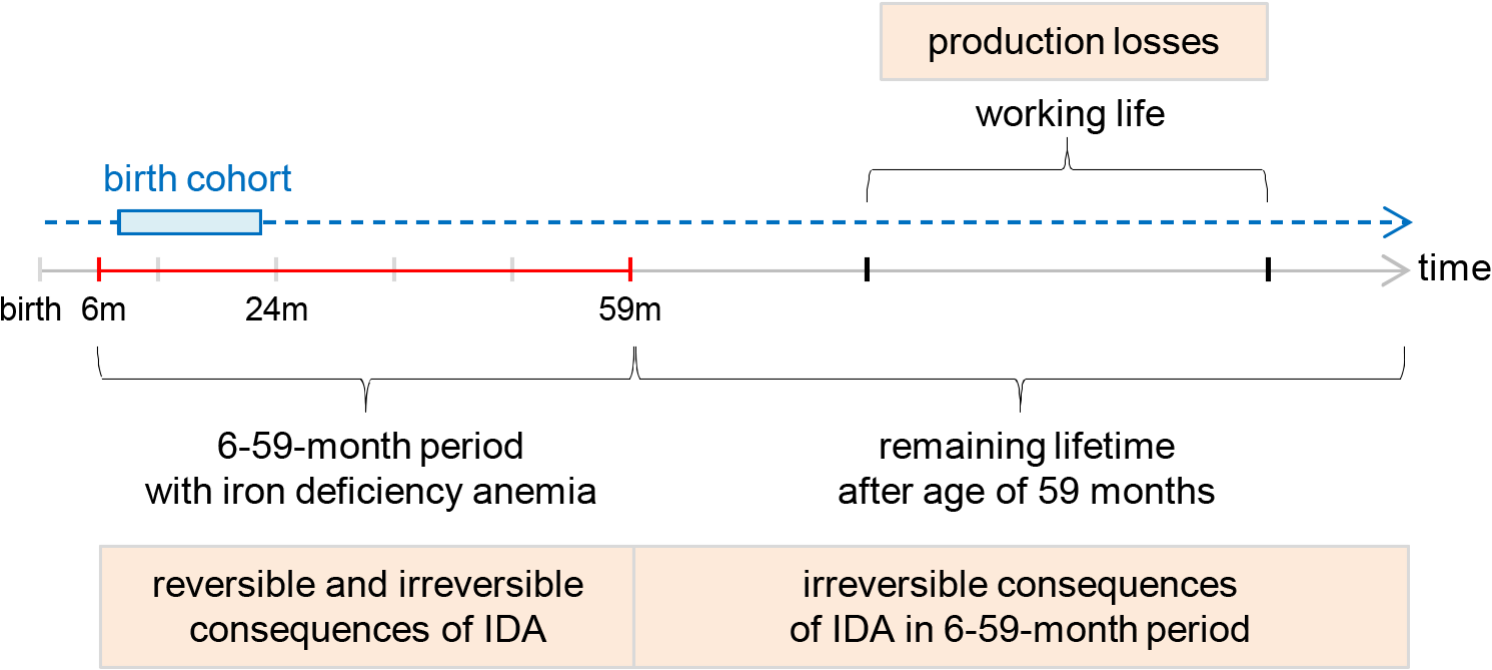
Temporary and permanent consequences of IDA in a birth cohort. The model estimates present value of the lifetime costs of iron deficiency anemia (IDA) in a birth cohort affected by IDA from 6 to 59 months of age. These costs include the irreversible effects of IDA on cognitive development, leading to lower incomes and lower quality of life in adult life.

In a fourth step we attribute the costs to the health consequences of IDA by multiplying the number of children affected by mild, moderate and severe IDA with the respective cost effects. This calculation takes into consideration that IDA leads to reversible as well as to irreversible health consequences. We model the costs of IDA as the lifetime-costs of a birth cohort of children, corresponding to all the children born in one year, in order to account for these irreversible consequences. Fig 2 illustrates this approach: The birth cohort is affected by IDA from the age of 6 to 59 months. The health and cost effects of this exposure to IDA arise within the 6–59-month time window as well as in the remaining lifetime of the birth cohort. These costs correspond to the present value of the social costs of IDA in a given year.

Intangible costs are calculated as DALYs lost [18] due to current illness, premature death and future permanent disabilities. We calculate the DALY losses according to the new WHO/GBD 2010 approach and compare the results with those obtained with the previous WHO/GBD 1996 approach [19, 20]. Future life years and DALYs lost are not discounted following the new WHO/GBD approach [21, 22]. We do not quantify DALY losses in monetary terms, as this is often criticized as ethically and methodologically questionable [23].

Production losses correspond to the future gross income losses due to impaired cognitive development caused by IDA in early childhood. Future production losses occur during work-life, starting at the age of labor force entry and ending with the end of work-life. Income losses are valued at the average Indian wage rate and not at the specific wages rates of the single SES. We thus implicitly assume some mobility across SES and that today’s children may migrate to urban areas in their future life. This assumption is confirmed by a recent report of the World Bank which finds a substantial economic mobility, both upward and downward, in India [24]. We also take account of future economic growth.

Wages are converted into US-dollars (USD) using an average 2013 exchange rate (current dollars). Future production losses are discounted to present value, as a dollar today is worth more than a dollar in 20 or 30 years, using a discount rate of 3% which is a widely accepted value in health economic evaluations [25].

Direct Medical costs are not considered in the model as anemia usually goes unnoticed and no treatment is provided.

Costs are calculated for the year 2013 and reported separately for the 6–23-month and 24–59-month age groups as costs of health consequences differ depending on the age at which the deficiency occurred. Aggregate information on the population and the economy has been drawn from the World Bank [26].

We run 2 types of sensitivity analysis (SA) in order to test the robustness of our results and understand the influence of single model parameters. First, we run a probabilistic SA, which allows us to establish a range of plausible model results by randomly varying all model parameters within predefined distributions and then running the model 10,000 times (see Wieser, Plessow [9] for details on the procedure). Second, we explore the influence of changes in the DALY methodology that were introduced by the GBD 2010 [8]. We analyze the effect of new disability weights and the new rules on discounting by calculating the intangible cost of IDA in early childhood according to both the old and the new methodology.

The model is implemented in R [27].

Ethics statement: The dataset used in this study was obtained from the International Institute for Population Sciences [10]. Review of this study from an institutional review board was not sought as the dataset is anonymous and available for public use with no identifiable information on the survey participants.

## Results

### Socio-economic patterns and child mortality

Table 1 shows the distribution of children in the birth cohort over all households stratified by SES and area of residence. The last column displays the total number of children in the birth cohort.

**Table 1:** Distribution of birth cohort by SES and area of residence.

	Share of SES (low to high) in total of children in area of residence (%)										total	% of
	1	2	3	4	5	6	7	8	9	10	(1,000)	total
rural	15.1	15.5	14.3	13.7	11.4	9.9	8.6	5.8	4.0	1.6	19&rsquo;501	73.8%
urban	1.5	2.7	3.3	4.9	6.7	9.2	13.8	17.5	19.5	20.9	6&rsquo;920	26.2%
total	11.6	12.1	11.4	11.4	10.2	9.8	10.0	8.9	8.1	6.7	26&rsquo;420	100.0%

Source: Own calculation on NFHS-3 [10] and 2011 Census data [5]

The table shows 2 clear patterns: First, the share of poor households is much higher in rural than in urban areas. In urban areas 72% of all children belong to SES 7–10, while in rural areas children are concentrated at the other end of the wealth scale with 58% of the households belonging to SES 1–4. Second, the number of children per household is higher in poorer households. Only 7% of all children live in the wealthiest 10% of households while 12% of all children live in a household belonging to the poorest 10%.

Table 2 summarizes the mortality rates, which have been calculated by quintiles, as the sample for deciles turned out to be too small to consistently estimate this rather rare event. Since mortality rates have decreased since 2005–06 we reduced the rates calculated on NFHS-3 data by 29.5%, which is the reduction observed in the World Bank data for the period from 2005 to 2013 [26].

**Table 2:** Yearly mortality rates per 1,000 children.

age group	area of residence	SES (low to high)					
		1+2	3+4	5+6	7+8	9+10	Overall
**6–23 months**	**rural**	9.5	7.4	5.4	3.4	1.3	5.3
	**urban**	11.5	8.1	5.5	3.8	2.9	4.4
**24–59 months**	**rural**	5.2	4.0	2.8	1.6	0.3	2.7
	**urban**	3.6	2.8	2.0	1.1	0.2	1.1

Source: Own calculation on NFHS-3 survey [10]

Mortality decreases as household wealth increases with a 3 to 7 times higher mortality in the poorest than in the wealthiest quintile of 6–23-month-old children. Mortality decreases from the 6–23 to the 24–59-month age-group and this decline is much more pronounced in wealthier households. Comparing mortality between rural and urban areas we find a lower mortality rate in rural areas within each wealth quintile but a higher overall mortality rate in rural areas. This apparently contradictory result is explained by the fact that children living in rural areas belong mainly to the poorer SES while children living in urban areas belong mainly to the wealthier SES (see Table 1).

### Prevalence of iron deficiency anemia

Table 3 reports the prevalence of IDA by severity, age-group and area of residence. The overall prevalence of IDA is 49.5% in the 6–23-month and 39.9% in the 24–59-month age-group, with mild IDA at 13.3% and 14.9%, moderate IDA at 33.8% and 24.0%, and severe IDA at 2.4% and 1.0% in the 6–23-month and 24–59-month age-groups, respectively. Moderate and severe IDA are thus higher in the 6–23-month age-group while mild IDA is higher in the 24–59-month age-group.

**Table 3:** Prevalence of IDA by severity, age-group and area of residence.

Age group	Area of residence	Iron deficiency anemia (%)			
		mild	moderate	severe	total
**6–23-months**	rural	13.3	34.8	2.3	50.4
	urban	13.2	30.8	2.6	46.6
	all	13.3	33.8	2.4	49.5
**24–59-months**	rural	15.2	24.9	1.0	41.1
	urban	14.3	21.4	1.0	36.7
	all	14.9	24.0	1.0	39.9

**Fig 3:**
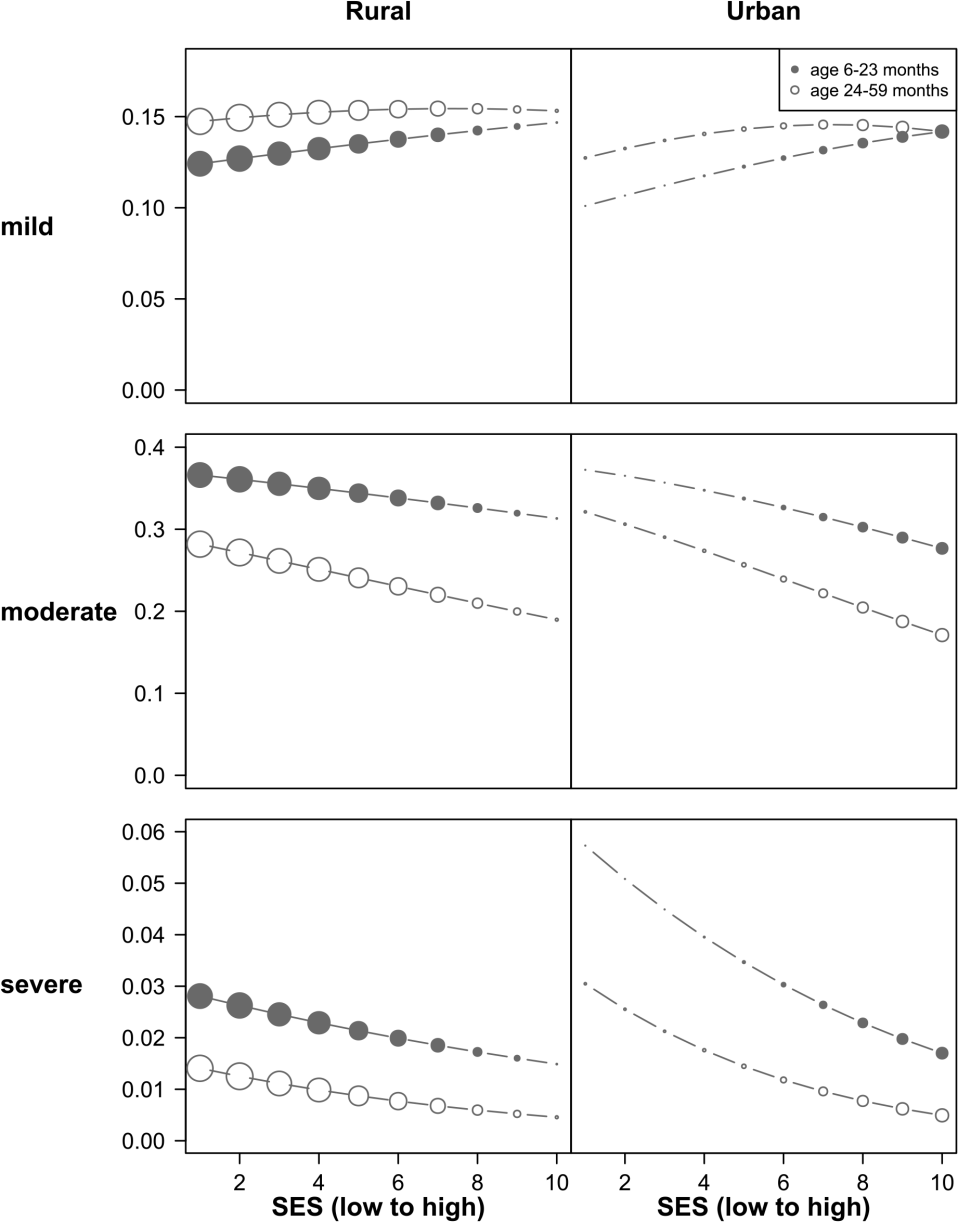
Prevalence of IDA by severity, age-group, SES and area of residence. Source: Own calculation based on NFHS-3 survey [10]. Mild IDA (Hb g/l 100–110); moderate IDA (Hb g/l 70–100); severe IDA (Hb g/l <70), size of bubbles corresponds to relative size of the population of the sub-group.

Fig 3 shows that moderate and severe IDA decrease as wealth increases, with prevalence of moderate and severe IDA being 0.5 and 4 times lower in the wealthiest 20% than in the poorest 20% of households respectively. The prevalence of mild IDA increases slightly as wealth increases, as children move from moderate and severe IDA to mild IDA, but this increase is not statistically significant.

The comparison between urban and rural households shows that the overall prevalence of mild, moderate and severe IDA is higher in rural than in urban areas. However, the distribution across SES shows that children living in poor urban households have higher risk of moderate and severe IDA than children living in equally poor rural households. The higher prevalence of severe IDA among the children of the urban poor is particularly striking. However, the overall number of iron-deficient children is considerably higher in rural than in urban areas as the large majority of poor households still lives in rural areas.

Finally, we did not find any major differences in the prevalence of IDA between boys and girls and therefore do not differentiate by gender in our analysis.

### Health and cost consequences of iron deficiency anemia

The model includes 3 different adverse health outcomes of IDA: First, IDA in 6–59-month-old children leads to impaired physical activity. This effect is temporary and ends as soon as the deficiency disappears. Second, severe IDA leads to increased mortality [28]. Third, moderate and severe IDA in children of 6–23 months leads to permanently impaired cognitive ability, which leads to a reduction in adult wage. In applying separate disability weights for impaired physical activity and cognitive impairment, we follow the approach by Stein et al. [29]. Table 4 gives an overview of the effect sizes of the health effects of IDA and the respective sources in the literature.

**Table 4:** Effect sizes and sources of health effects of IDA.

Health Consequence	Severity of IDA	Age-groups affected	Size of health effect of IDA	Disability weights of health consequence (WHO/GBD [18])	Permanent irreversible effect
**Impaired physical activity**	mild	both	effects only in terms of DALYs	0.005	no
	moderate	both	effects only in terms of DALYs	0.058	no
	severe	both	effects only in terms of DALYs	0.164	no
**All-cause mortality**	severe	both	RR of mortality 2.19 [28]	1	yes
**Cognitive impairment**	moderate	6–23-months	Cog. score in adults: -0.6 SD [3]	0.0078	yes
	severe	6–23-months	Cog. score in adults: -0.6 SD [3]	0.031	yes

**Fig 4:**
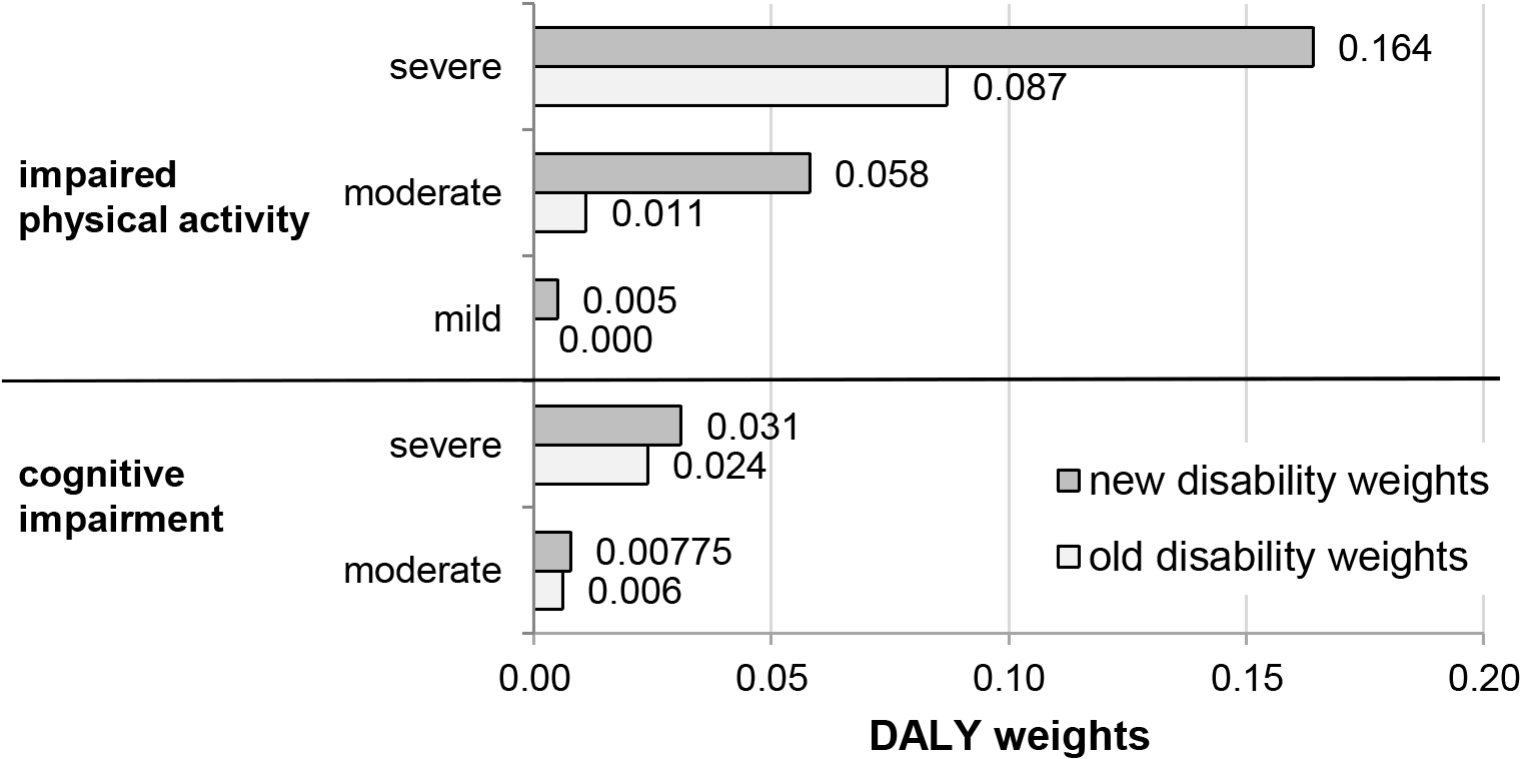
Old and new disability weights. Disability weights according to GBD 1996 methodology [19] and GBD 2010 methodology [18] and adaptations according to Stein et al. [29].

We use the new disability weights according to the GBD 2010 [18]. These new weights are based on large population surveys, while the previous disability weights were based on expert opinion [18, 19]. All disability weights for the health consequences of IDA have increased from the old to the new GBD version (Fig 4) and these increases are particularly strong for impaired physical activity, which, according to the GBD 2010, is now also a consequence of mild anemia. The increase in disability weights for cognitive impairment is less pronounced but equally important due to its irreversible nature. As the GBD 2010 does not provide a weight for cognitive impairment due to moderate anemia, we calculate a corresponding weight by increasing the GBD 1996 weight by the same proportion as the disability weight for cognitive impairment due to severe anemia (+29.2%).

A further major change occurred in the discounting of future DALYs: while there was an option for discounting in the GBD 1996 version, it has been eliminated from the new GBD 2010 methodology [18].

Table 5 displays the values and sources of additional economic parameters required for the calculation of production losses and DALYs in the model. Most parameters are based on World Bank data [26]. Future income growth is derived from OECD data [30] and daily wages have been obtained from the International labour organization (ILO) [31].

**Table 5:** Economic model parameters.

Economic Parameters	Value	Source
Life expectancy	66.2 years	World Bank [26]
Monetary discount rate	3%	Smith & Gravelle [25]
DALY discount rate	0%	GBD 2010 [8]
Future income growth	3%	Own calculation based on [30]
Exchange rate (INR / USD)	59	World Bank [26]
Begin of working life	15	World Bank [26]
End of working life	64	World Bank [26]
Workforce participation	57.90%	World Bank [26]
Unemployment rate	3.40%	World Bank [26]
Daily wage	5.3 USD	Own calculation based on ILO data [31]

### Social costs of iron deficiency anemia

Total lifetime costs of IDA between the ages of 6 and 59 months in a birth cohort in 2013 of Indian children represent intangible costs of 8,321,254 DALYs and production losses of 24,001 million US-dollars in 2013. For ease of interpretation, we can transform DALYs to potential lives lost by dividing the number of DALYs by the average life expectancy. The 8,321,254 DALYs thus correspond to the impressive number of 125,699 complete lifespans lost in India every single year due to IDA.

Table 6 reports the detailed results by age-group, area of residence, type of social cost, and time dimension of cost (current, future, mortality). The distinction between current and future costs is important, as future costs are caused exclusively by IDA in 6–23-month-old children due to the irreversible cognitive impairment triggered by moderate and severe IDA in this age-group. Future losses due to impaired cognitive development in early childhood dominate both production losses (98% of total) and intangible costs (66.4% of total). Future effects are much larger than current effects as the birth cohort will live most of its life after the age of 5.

**Table 6:** Social cost of IDA by age-group and time dimension of cost in 2013.

		Production losses			Intangible Costs			
		(million USD)			(in 1000 DALYs)			
		Future	Mortality	Total	Current	Future	Mortality	Total
**6–23 months**	Rural	17,755	281	18,036	675	4,139	345	5,159
	Urban	5,730	81	5,811	224	1,389	100	1,713
	All India	23,485	362	23,847	899	5,528	444	6,871
**24–59 months**	Rural		134	134	962		160	1,122
	Urban		20	20	304		23	327
	All India		154	154	1,266		184	1,450
**both**	Rural	17,755	415	18,170	1,637	4,139	505	6,281
	Urban	5,730	101	5,831	528	1,389	123	2,040
	All India	23,485	516	24,001	2,165	5,528	628	8,321

Current costs occur at 6–59-months of age; future costs occur after the age of 5 years.

The results show that rural areas bear the highest burden of IDA. With a 2.8 times larger population, rural areas have 3.1 times higher intangible costs and production losses than urban areas.

**Fig 5:**
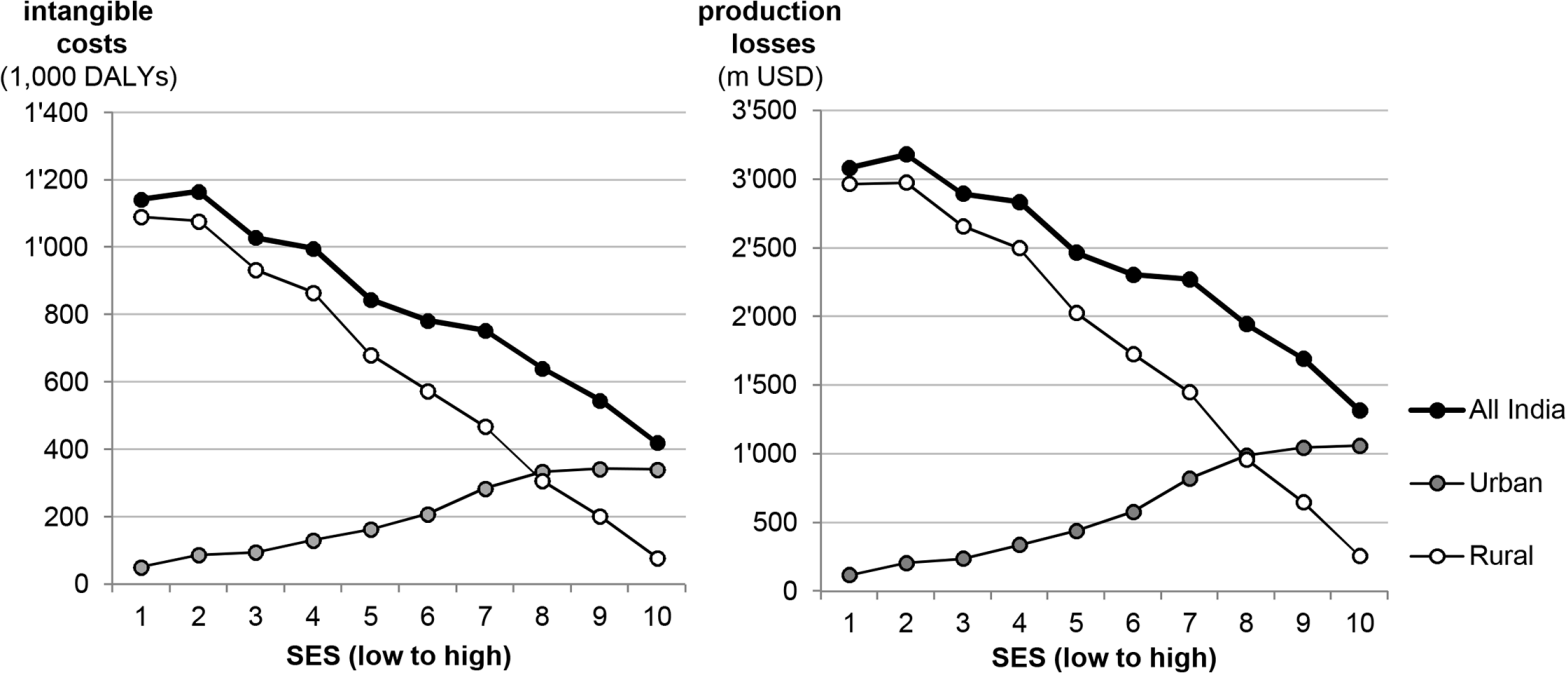
Intangible costs and production losses due to IDA by SES. Distribution of costs across SES. Households are split into deciles of equal size for the whole country.

Total costs of IDA differ substantially across SES with 2.4 times higher intangible costs in the poorest than in the wealthiest quintile and 2.1 times higher production losses (Fig 5). The marked differences between urban and rural areas in Fig 5 are mainly due to the higher number of rural households in the poorest SES and of urban households in the wealthier SES. Overall losses of IDA are high even in wealthy households.

### Sensitivity analysis

**Fig 6:**
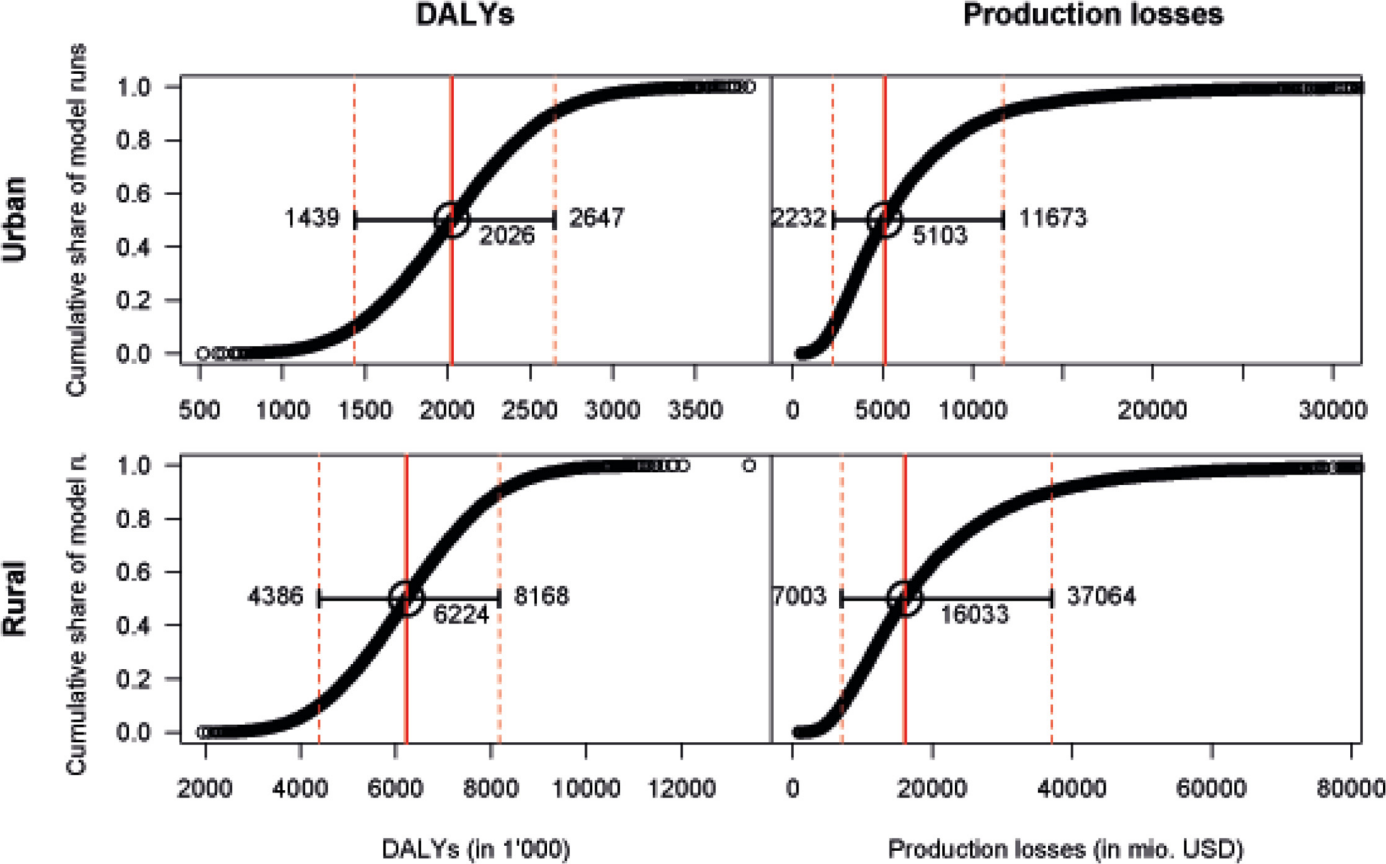
Multivariate sensitivity analysis by type of social cost and area of residence. Distribution of ordered results from 10,000 model runs with input parameters varying randomly according to predefined distributions. The curves show the share of all model runs that resulted in costs of a given size or lower. The solid vertical line shows the median result of all model runs. The dashed lines mark the values between which 80% of all model runs lay.

We assess the overall uncertainty of our results with a multivariate probabilistic SA by drawing model parameters from an appropriately parameterized distribution. Fig 6 shows the ordered results of 10,000 model runs for total intangible costs and total production losses. The figures show the share of all model runs that resulted in a cost at or below a given value. Uncertainty in intangible costs is relatively limited with 80% of all cases in a range between 1,4 m and 2,6 m DALYs in urban and between 4,4 and 8,2 m DALYs in rural India. Production losses show larger variation with 80% of all cases between 2,232 and 11,673 m USD in urban and between 7,003 and 37,064 m USD in rural India. Upward variation is substantially larger due to the uncertainty regarding the future development of the Indian economy.

**Fig 7:**
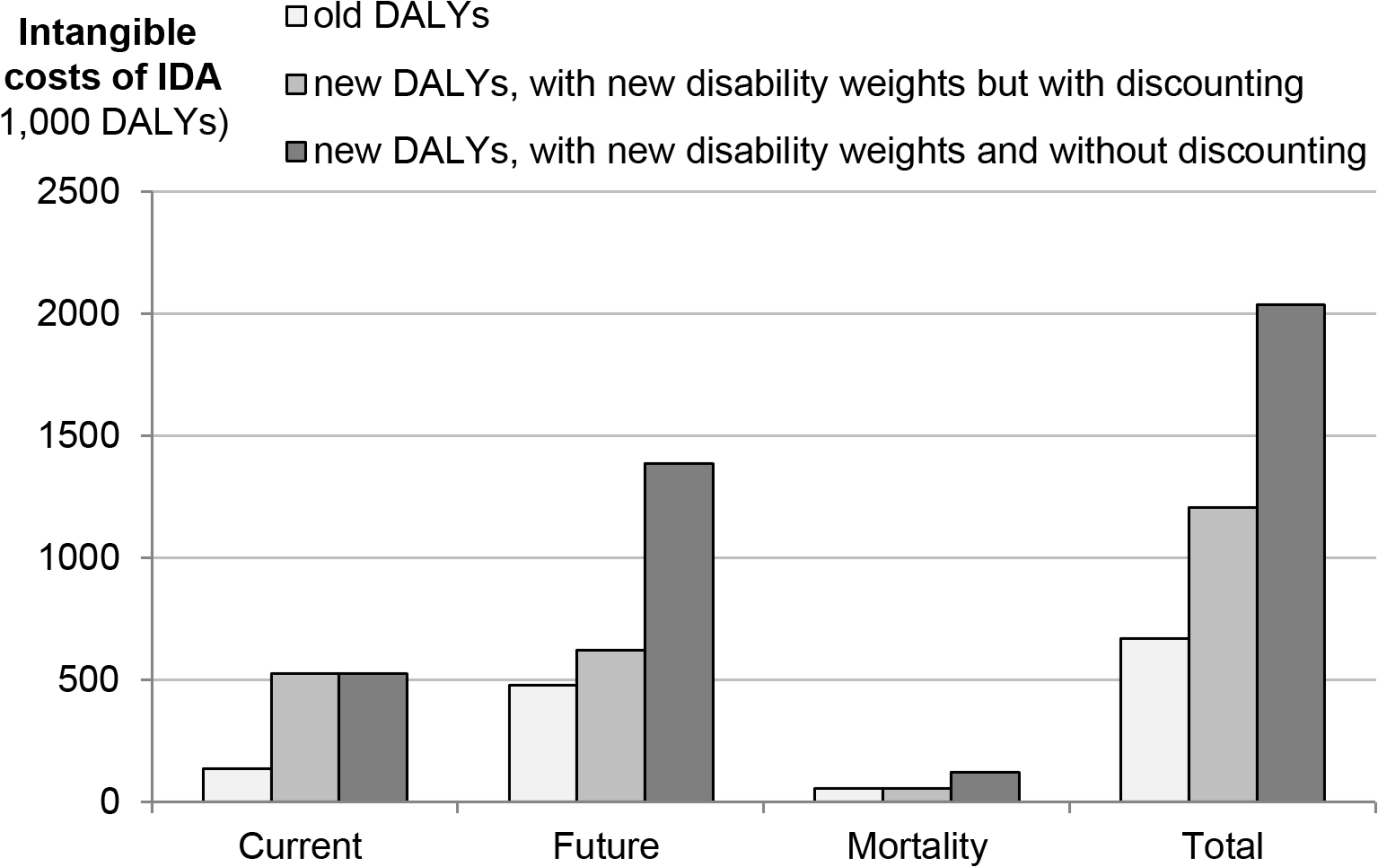
Comparison of DALY calculation rules for urban India.

In a second SA we explore the effect on intangible costs of the changes in the new GBD-method, which include the abolition of discounting of future DALY losses and increased disability weights for the health effects of IDA. Fig 7 shows that the new method leads to a substantial increase of DALY losses. The introduction of the new disability weights leads to a nearly fourfold increase of current losses and a slight increase of future losses. DALY losses due to increased mortality are not affected, as the disability weight of death does not change. Elimination of discounting has no effect on current losses but increases future losses and losses due to mortality more than twofold. The strong effect of discounting is due to the fact that 75% of intangible costs occur in the future. Overall intangible costs of IDA in 6–59-month-old children triple with the introduction of the new GBD-method.

## Discussion

The prevalence of IDA is still extremely high in 6–59-month-old Indian children with almost every second child suffering from some degree of IDA. Our results indicate that total lifetime costs of IDA in a birth cohort affected by IDA between the age of 6 and 59 months amount to intangible costs of 8.3 m DALYs and production losses of 24,001m USD in 2013. Intangible costs correspond to 125,699 complete lifespans lost, or 0.48% of the healthy life years of the birth cohort, and production losses correspond to 1.3% of gross domestic product. The SA suggests that our results are relatively robust and that the intangible costs of IDA have been substantially underestimated with the previous WHO/GBD methodology.

Both intangible costs and production losses are dominated by future losses due to impaired cognitive development, which is caused by IDA between the age of 6 and 23 months. The social costs of IDA arise in all socio-economic groups, with the largest share occurring in poor rural households but even wealthy urban households carrying a considerable share.

Our estimate can be considered as the lower bound of the social costs of IDA in 6–59-month-old children because it does not include a number of possible cost consequences: 1) We estimate production losses as future income losses without considering non-market production, such as subsistence agriculture, which may be especially important in rural areas. Poor households will also be particularly affected by income losses as a wage reduction may have disastrous effects for a subsistence farmer, who produces little more than what is required for bare survival, while wealthier individuals will suffer less from a similar decrease. 2) We do not consider direct medical costs due to IDA. Severe IDA is a serious illness and some children certainly receive treatment. However, we lack data to estimate the extent of these medical costs. Affected poor households are likely to incur catastrophic health expenditures due to required out-of-pocket payments[32].

Our results appear plausible when compared with previous studies. The Institute of Health Metrics and Evaluation (IHME) attributes 3.3m DALYs to iron deficiency in 1–4-year-old children [33], a result that at first sight appears to be substantially below our result of 8.3m DALYs for 6–59-month-old children. This difference is, however, explained by the differences in the methodological approach and in the health consequences considered. We employ an incidence-based approach which considers the lifetime impact of impaired cognitive development due to IDA in early childhood, and includes the impact of all-cause mortality. Our estimate of the direct effects of iron deficiency amounts to 60% of the value reported by the IHME and is thus a more conservative estimate.

Our results show that although IDA has been on the political agenda for the past 40 years [34], it is still a massive problem in India. IDA is essentially a consequence of the low level of bioavailable iron in the diet of the children and their mothers [35]. Indian diets are low in bioavailable iron due to phytates, which inhibit the absorption of iron, and low ascorbic acid / iron ratios, which facilitate the absorption of iron [23].

Despite the strong economic growth in India in the last two decades, there has been little improvement in the iron intake. Actually household expenditures on nutrition did not increase with income while nutritional deficiencies persist [36].

Many interventions appear to be ineffective and do not focus on the age-group of children below the age of 2, which is most in need [37, 38]. In theory, public programs should provide every child between the age of 12 and 59 months with iron supplements, but in reality program coverage is very low at around 3–4% [2, 39].

IDA has particularly severe socio-economic consequences for poor households as it leads to a health-based poverty trap [37, 40]. Due to the impact of IDA on cognitive development, many children will not be able reach their full potential and become poor parents in their later life. The combination of IDA and other nutritional deficiencies with a weak education system and an inadequate provision of health care services is a key obstacle to the economic development of India [41].

There is an urgent need for more frequent data on the health and nutritional status of the Indian population, in particular of children and women at childbearing age. The 10-year-intervals of the NFHS survey are too long for a timely monitoring of crucial public health issues.

Our study has a number of limitations: 1) We make a series of assumptions on the long-term development of important model parameters such as the future economic growth rate, which crucially affect the magnitude of production losses. However, we always apply conservative estimates of these parameters and carry out an extensive multivariate SA. 2) We do not take account of the intergenerational effects of malnutrition, which have been the focus of recent research (see for example [42–44]). However these findings do not allow us to attribute specific effect sizes to specific types of malnutrition occurring in specific age-groups and can thus not be considered in our model.

## Conclusion

IDA in 6–23-month-old children remains a major health problem in India and its social costs remain extremely high, both in terms of DALYs and income losses. There is an urgent need for effective interventions capable of improving the nutritional status of children under 5 and in particular of the 6–23-month-olds. The results may have important implications for the conception and targeting of future policies aimed at the reduction of IDA prevalence.
